# Water-splitting-based, sustainable and efficient H_2_ production in green algae as achieved by substrate limitation of the Calvin–Benson–Bassham cycle

**DOI:** 10.1186/s13068-018-1069-0

**Published:** 2018-03-19

**Authors:** Valéria Nagy, Anna Podmaniczki, André Vidal-Meireles, Roland Tengölics, László Kovács, Gábor Rákhely, Alberto Scoma, Szilvia Z. Tóth

**Affiliations:** 1 Institute of Plant Biology, Biological Research Centre of the Hungarian Academy of Sciences, Szeged, Temesvári krt. 62, 6726 Szeged, Hungary; 2 Institute of Biochemistry, Biological Research Centre of the Hungarian Academy of Sciences, Szeged, Temesvári krt. 62, 6726 Szeged, Hungary; 3 Department of Biotechnology, University of Szeged, Közép fasor 52, 6726 Szeged, Hungary; 4 Institute of Biophysics, Biological Research Centre of the Hungarian Academy of Sciences, Szeged, Temesvári krt. 62, 6726 Szeged, Hungary; 5 Center for Geomicrobiology, Aarhus University, Ny Munkegade 116, 8000 Aarhus, Denmark

**Keywords:** Biohydrogen, Calvin–Benson–Bassham cycle, *Chlamydomonas reinhardtii*, Hydrogenase, Oxygen absorbent, Oxygen evolution, Photosynthesis

## Abstract

**Background:**

Photobiological H_2_ production has the potential of becoming a carbon-free renewable energy source, because upon the combustion of H_2_, only water is produced. The [Fe–Fe]-type hydrogenases of green algae are highly active, although extremely O_2_-sensitive. Sulphur deprivation is a common way to induce H_2_ production, which, however, relies substantially on organic substrates and imposes a severe stress effect resulting in the degradation of the photosynthetic apparatus.

**Results:**

We report on the establishment of an alternative H_2_ production method by green algae that is based on a short anaerobic induction, keeping the Calvin–Benson–Bassham cycle inactive by substrate limitation and preserving hydrogenase activity by applying a simple catalyst to remove the evolved O_2_. Cultures remain photosynthetically active for several days, with the electrons feeding the hydrogenases mostly derived from water. The amount of H_2_ produced is higher as compared to the sulphur-deprivation procedure and the process is photoautotrophic.

**Conclusion:**

Our protocol demonstrates that it is possible to sustainably use algal cells as whole-cell catalysts for H_2_ production, which enables industrial application of algal biohydrogen production.

**Electronic supplementary material:**

The online version of this article (10.1186/s13068-018-1069-0) contains supplementary material, which is available to authorized users.

## Background

Among the potential, renewable energy-converting technologies, photobiological H_2_ production stands out as an appealing choice, because it is carried out by microorganisms in an aqueous environment, possibly without arable land requirement. Biohydrogen produced by algae may become a genuinely carbon-free energy carrier because, as opposed to bioethanol and biodiesel production, upon the combustion of H_2_ only water is produced (reviewed by [1]).

**Fig. 1:**
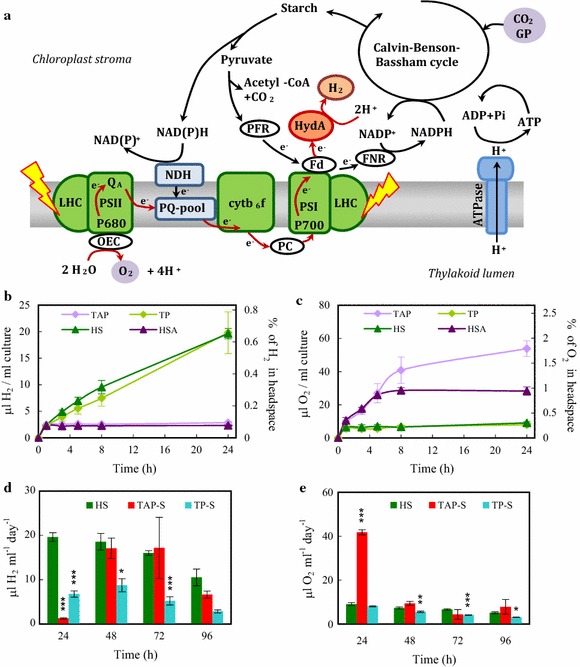
H_2_ production in *Chlamydomonas reinhardtii*. **a** Schematic presentation of the photosynthetic electron transport chain in green algae. Solar energy is captured by the light-harvesting complexes (LHC) of photosystem II and I (PSII and PSI). Electrons extracted from water by the oxygen-evolving complex (OEC) of PSII are transferred to the photosynthetic electron transport chain via the plastoquinone (PQ)-pool, the cytochrome b_6_f complex (cyt b_6_f), plastocyanin (PC), PSI and ferredoxin (Fd). From Fd, electrons can be transferred by the ferredoxin-NADP^+^ oxidoreductase (FNR) to NADP^+^ or to the hydrogenase (HydA; for clarity, oxygen-dependent alternative pathways are not shown). H^+^ accumulated in the thylakoid lumen are used for ATP production via ATP synthase. The ATP and NADPH generated during primary photosynthetic processes are consumed for CO_2_ fixation in the Calvin–Benson–Bassham (CBB) cycle, which produces sugars and ultimately starch. When cultures are grown in the presence of acetate, glycerate 3-phosphate (GP) may also feed the CBB cycle. Hydrogenases are expressed under anoxic conditions; upon illumination, significant H_2_ production may occur. The water-oxidation dependent pathway of H_2_ production is denoted with red line. Depending on the conditions, starch degradation may also contribute to H_2_ production either via the NAD dehydrogenase (NDH) complex, PSII-independent pathway, or via pyruvate-Fd-oxidoreductase (PFR). **b** H_2_ production in CC124 *Chlamydomonas* cultures (50 µg chl (a + b)/ml, at 320 µmol photons/m^2^/s) as determined in the headspaces of sealed cultures using gas chromatography during 24 h in acetate-containing (TAP, HSA) and acetate-free media (TP, HS) following dark anaerobic incubation (4 h darkness with 3 × 10 min N_2_ flushing). In the right *Y* axis, the percentage of H_2_ in the headspaces of the cultures are shown. Time point 0 indicates the time when the cultures were transferred to the light. **c** Net O_2_ production under the same conditions as in **b**. **d** Daily H_2_ production in sulphur-containing HS medium of cultures subjected to dark anaerobic incubation and in cultures transferred to sulphur-free acetate-containing (TAP-S) or acetate-free (TP-S) media. All the alga cultures (HS, TAP-S, TP-S) were illuminated continuously and flushed every 24 h with N_2_ after determining the gas concentrations in the headspaces of the sealed bottles to avoid excessive H_2_ accumulation (cf. [7]) and overpressure in the headspace. **e** Net O_2_ production under the same conditions as in **d**. Mean values (± SEM) are each based on 6 biological replicates. Statistical significance levels are presented relative to the *Chlamydomonas* culture subjected to dark anaerobic incubation in HS medium as *p < 0.05, **p < 0.01, ***p < 0.001

[Fe–Fe]-type hydrogenases found in green algae are one of the most active molecular catalysts known for H_2_ production. The green alga *C. reinhardtii* has two [Fe–Fe]-type hydrogenase paralogues, called HydA1 and HydA2; the turnover rate of the major form, HydA1, is several thousands per second, approx. 100-fold higher than that of other type of hydrogenases [2]. Hydrogenases are located in the chloroplasts stroma, at the acceptor side of photosystem I (PSI, Fig. 1a). They may receive electrons from various sources, of which photosynthetic linear electron transport may be the most prominent one. Starch degradation can also feed electrons into the electron transport via the NAD dehydrogenase (NDH) complex, independently of photosystem II (PSII) activity. The third pathway for H_2_ production includes pyruvate oxidation through pyruvate–ferredoxin-oxidoreductase (PFR; reviewed by [1]).

Hydrogenases become highly expressed under hypoxic conditions established for instance during the night when the microbial community consumes the available O_2_; upon illumination, electrons from ferredoxin (FDX) in PSI are transferred to the hydrogenases. Out of the 13 types of FDXs found in *Chlamydomonas* [3], FDX1 and FDX2 contribute to H_2_ production [4, 5]. The midpoint redox potential of the major photosynthetic FDX1 is −0.398 V enabling efficient electron donation to HydA [6, 7].

Hydrogenases catalyze the reduction of protons, thereby supporting an alternative electron transport to prevent the over-reduction of the photosynthetic electron transport chain upon dark-to-light transitions. By this reaction, hydrogenases also promote the light-induced increase of stromal pH necessary for the activation of the Calvin–Benson–Bassham (CBB) cycle, ultimately supporting ATP formation (reviewed by, e.g. [8, 9]). Thus, hydrogenases play an essential role upon the induction of photosynthesis in green algae [10]. Once the electron transport is fully functional, the O_2_ evolved by PSII reacts with hydrogenases, leaving an inactive [4Fe–4S] subcluster state [11] and with O_2_ also inhibiting *HYDA1* gene expression [12, 13].

Owing to the high theoretical efficiency of converting the energy of sunlight into chemical energy [2], photobiological H_2_ production by green algae has been studied for decades with O_2_ sensitivity of the hydrogenases being a major hurdle in reaching commercial viability. Engineering O_2_-insensitive hydrogenases has been attempted with moderate success (e.g. [14]). Alternatively, hypoxia can be established by downregulating PSII activity, most commonly by sulphur deprivation (e.g. [15], reviewed recently by [16]). However, this method is unspecific and results in the degradation of photosynthetic complexes and cell death on the timescale of days [17]. H_2_ production induced by sulphur deprivation is also strongly dependent on acetate or other organic carbon sources, meaning that in a narrow sense it is not a photoautotrophic process [18, 19], and has a low inherent energy conversion efficiency [20]. As it stands, it is very unlikely that sulphur deprivation will represent a viable procedure for industrial H_2_ production [20]. Nitrogen [21], phosphorous [22] and magnesium starvations [23] have been also attempted, but pose similar problems to sulphur deprivation.

H_2_ production can also be induced in nutrient-replete Tris–acetate–phosphate (TAP) medium, by incubating *Chlamydomonas* cultures in the dark for a few hrs in O_2_-free atmosphere and exposing them to relatively low light. Under these conditions, H_2_ production is temporary, because the evolved O_2_ inhibits the hydrogenases within a few hrs (reviewed by [24]). By incubating the cultures under very low light conditions, a balanced O_2_ evolution and respiration can be established, resulting in low-yield H_2_ production lasting for several days [25]. This type of H_2_ production depends largely on photosynthesis and partly on fermentative processes [25]. The yield of H_2_ production can be increased by enhancing acetate respiration [26], which, however, represents a severe loss of efficiency, as for each H_2_ produced an equivalent amount of substrate is respired [27]. A direct and efficient conversion of solar energy to H_2_ in a carbon–neutral way is required for commercial viability [20, 28]. Anaerobiosis-induced H_2_ production has been observed in photoautotrophic (i.e. acetate-free, CO_2_-supplemented) cultures as well, in which H_2_ production lasted for several days at low light [29, 30]), although with a very low efficiency as compared to the widely used sulphur-deprivation method in TAP medium. Thus, in recent protocols applying anaerobic incubation the CBB cycle was operational, even though the competing nature of CO_2_ assimilation and H_2_ production was already shown by Gaffron and Rubin [31] upon discovering the photoproduction of H_2_ by green algae.

The present approach to improve photobiological H_2_ production stem from the ultimate physiological role of the highly efficient hydrogenases found in green algae (Fig. 1a). Their primary function is to serve as a safety valve [32], i.e. alternative electron acceptors upon the induction of photosynthesis in hypoxia [10, 33]; once the photosynthetic apparatus is fully functional, the hydrogenases become inactive by the evolved O_2_. We show here that by preventing the activation of the CBB cycle in the light via substrate limitation (i.e. by omitting CO_2_ or acetate), the photosynthetic electron transport chain remains largely reduced, resulting in low O_2_ evolution and high H_2_ production lasting for several days. The additional application of an iron-based O_2_ absorbent resulted in yields that are significantly greater than for the standard sulphur deprivation procedure.

## Results

### Dark anaerobic incubation of Chlamydomonas cultures followed by continuous illumination in acetate-free media results in substantial H_2_ production lasting for several days

Dark anaerobic incubation treatments to induce hydrogenase expression were carried out with the CC124 strain of *Chlamydomonas reinhardtii*, because it is a relatively efficient H_2_ producer and has been successfully used under various conditions (e.g. [34–37]). After growing the cultures for 3 days in TAP medium, the cells were transferred to culture media with (TAP, HSA) or without acetate (TP, HS) with a chlorophyll (chl) (a + b) content set at 50 µg/ml. Hydrogenase expression was induced by a 4-h dark anaerobic incubation during which N_2_ flushing was applied to remove both O_2_ and CO_2_. The high chl (a + b) content was set to facilitate the establishment of anaerobiosis, PQ-pool reduction and high hydrogenase expression [38]. For a general scheme of the experimental setup, see Additional file 1: Fig. S1.

As opposed to most earlier studies in nutrient-replete conditions, we subjected the cultures to relatively high light intensities (320 µmol photons/m^2^/s provided by white fluorescent tubes) after the dark anaerobic induction. Upon light exposure, H_2_ production (approx. 2 µl H_2_/ml culture) was observed during the first hour in all growth media. In acetate-containing media (TAP and HSA), prolonged illumination did not result in further H_2_ production, whereas in acetate-free media (HS, TP) H_2_ production continued (approx. 20 µl H_2_/ml culture in 24 h, Fig. 1b). The amount of O_2_ in the headspace of the vials rapidly accumulated in the presence of acetate (Fig. 1c), whereas in its absence the O_2_ concentration remained at a low level (approx. 9 µl O_2_/ml culture was produced, corresponding to approx. 0.3% O_2_ in the headspace). As cultures kept in HSA or TAP had low H_2_ yields and both TP and HS cultures produced large amounts of H_2_, the increase in H_2_ production efficiency was attributed to the absence of acetate. Therefore, in the following experiments, we opted for the HS media, as it is commonly used as a minimal media for studying algal physiology (e.g. [39]).

Next, we compared the efficiency of H_2_ production following a dark anaerobic induction in HS medium (performed as described above) with the classical sulphur deprivation method that is largely dependent on acetate [18, 40]. In cultures induced by dark anaerobiosis in HS media approx. 20 µl H_2_/ml (= 16.32 nmole H_2_/µg chl (a + b)) was produced during the first 24 h (Fig. 1d). The produced gases were removed every 24 h by N_2_ flushing after determining the amount of gases produced to promote the establishment of hypoxia, and to avoid a high H_2_ partial pressure [7] and overpressure in the headspace of the cultures. Using this method, the total H_2_ production in HS media was approx. 65 µl H_2_/ml culture in 96 h (Fig. 1d, sum of the green columns). In the case of sulphur deprivation (TAP-S, Fig. 1d), H_2_ production was low during the first 24 h, with the maximum H_2_ output detected after 48 and 72 h; the total production was approx. 42 µl H_2_/ml culture in 96 h (Fig. 1d, sum of the red columns). This productivity is consistent with earlier results obtained with the CC124 strain when using sealed flasks (e.g. [37, 41]), but it is below the yields attained with improved photobioreactor (PBR) systems on the same timescale (approx. 200 µl H_2_/ml culture in 96 h, [42]). When acetate was omitted from the media of cultures subjected to sulphur deprivation (TP-S, Fig. 1d), the amount of H_2_ produced was strongly diminished, corroborating the notion that the sulphur deprivation procedure is acetate-dependent [18, 40].

After 24 h of illumination, approx. 9 µl O_2_/ml was accumulated in the HS and TP-S cultures, whereas 40 µl O_2_/ml culture accumulated in the headspaces of sulphur-deprived samples (corresponding to approx. 1.3% O_2_, Fig. 1e).

These results demonstrate that anaerobic induction of hydrogenases in minimal (i.e. acetate-free) media can result in sustained H_2_ production of several days, with yields higher than in TAP-S media at equivalent culture conditions.

### Origin and regulation of H_2_ production in acetate-free media

Since the H_2_ production induced by anaerobic incubation is carried out in acetate-free media, and no sulphur deprivation is involved that would induce starch accumulation [17], electrons supporting H_2_ production likely originated directly from water (Fig. 1a). To test this hypothesis, we first measured the starch content throughout the experiment (Additional file 2: Fig. S2). The starch content of control, aerobic cultures was about 0.25 mmol/l, typical of aerobic, non stressed samples [43]. Following the 4-h dark-incubation starch content decreased to about 0.05 mmol/l. Upon transfer to the light (i.e. the start of H_2_ production) the starch content increased but did not reach the control level. Between 24 and 96 h of H_2_ production, there was no major change in the starch content, indicating that starch degradation does not contribute significantly to H_2_ production.

**Fig. 2:**
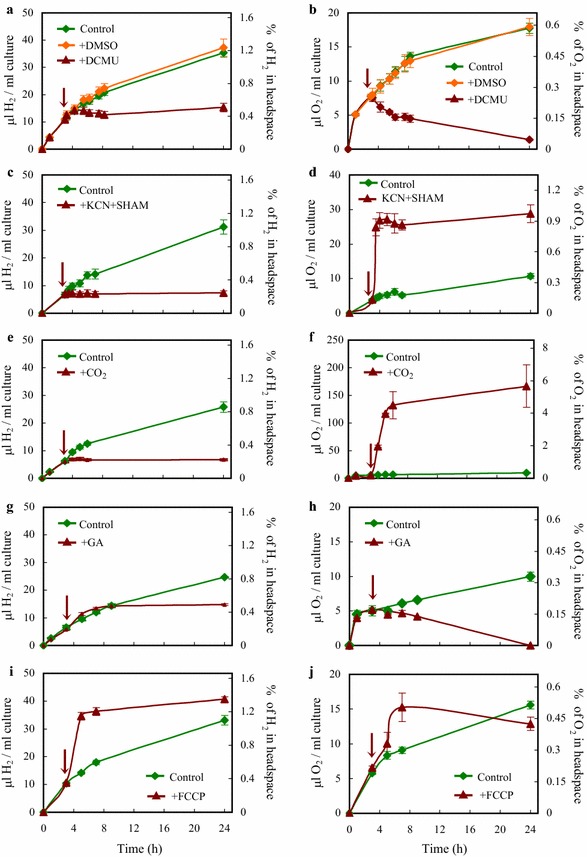
Photosynthetic electron transport during H_2_ production of *Chlamydomonas* in acetate-free HS medium induced by dark anaerobic incubation. **a**, **b** H_2_ and net O_2_ productions upon the addition of the PSII inhibitor DCMU, and DMSO, used as a control. **c**, **d** H_2_ and net O_2_ productions upon the addition of the respiratory inhibitors potassium cyanide (KCN) and salicylhydroxamic acid (SHAM). **e**, **f** H_2_ and net O_2_ productions upon the addition of 2% CO_2_. **g**, **h** H_2_ and net O_2_ productions upon the addition of the CBB cycle inhibitor glycolaldehyde (GA). **i**, **j** H_2_ and net O_2_ productions upon the addition of and the ionophore carbonylcyanide *p*-triflouromethoxyphenylhydrazone (FCCP). The time points at which the various treatments were applied are indicated by arrow. In the right *Y* axis, the percentage of the corresponding gases in the headspaces of the cultures are shown. All experiments were carried out at 320 µmol photons/m^2^/s following the 4-h dark anaerobic incubation (0 time point). Mean values (± SEM) are each based on 4 or 5 biological replicates

Next, we treated the cells with 3-(3′,4′-dichlorophenyl)-1,1-dimethylurea (DCMU), which irreversibly binds to the Q_B_ site in PSII [44]. Upon the application of DCMU, the amount of O_2_ in the headspace decreased and H_2_ production ceased with a slight delay (Fig. 2a, b), possibly due to degassing of H_2_ from the media. DMSO-treatment used as a control resulted in no discernable effect on H_2_ and O_2_ productions relative to the control (Fig. 2a, b). These results demonstrate the direct dependence of H_2_ production on PSII electron transport and water-splitting, and suggest that the contribution of cellular respiration to H_2_ production is minor.

To test whether respiration contributes to the establishment of hypoxic conditions, we treated the cultures with potassium cyanide (KCN, inhibiting the terminal oxidase) and salicylhydroxamic acid (SHAM, inhibiting the alternative oxidase). Upon their addition, the O_2_ concentration in the headspace of the cultures increased suddenly from approx. 0.3 to 0.9%, and remained at this level thereafter (Fig. 2d). Upon the application of the respiratory inhibitors, H_2_ production ceased (Fig. 2c), probably as a result of the increased O_2_ concentration.

In *Chlamydomonas*, acetate assimilation may occur via the tricarboxylic acid cycle and the glyoxylate cycle, which are metabolically linked to gluconeogenesis and the oxidative pentose phosphate pathway [45, 46]. The released CO_2_ and glycerate 3-phosphate feed the CBB cycle for which the reducing power generated by the photosynthetic electron transport is also utilized (Fig. 1a), as indicated by the extreme light sensitivity of various Rubisco mutants [47, 48]. We hypothesized that in the absence of acetate and CO_2_, the CBB cycle is mostly inactive and the electrons originating from the photosynthetic electron transport chain are transferred to the hydrogenases. To ascertain about this, we added 2% CO_2_ (v/v) into the headspace of the cultures. As a result, H_2_ production ceased, whereas O_2_ production strongly increased (Fig. 2e, f). Concomitantly, the amount of CO_2_ in the headspace of the cultures diminished rapidly and after 24 h no CO_2_ could be detected (Additional file 3: Table S1).

When the CBB cycle inhibitor glycolaldehyde (GA) was added, H_2_ production was unchanged during the initial 9 h, whereas the O_2_ concentration in the headspace decreased (Fig. 2g, h). By the 24th hour, H_2_ production ceased and no O_2_ could be detected, which is most probably due to a side-effect of GA on photosynthesis [49]. These results demonstrate that the CBB cycle activity, which would compete for electrons with the hydrogenases, must be low. There may be some remaining activity only at the beginning of illumination explaining the transitional increase in starch content following the dark-anaerobic incubation (first 24 h in the light, Additional file 2: Fig. S2).

Upon low substrate availability for the CBB cycle, the consumption of ATP and NADPH is decreased, triggering adjustments of the light reactions to prevent damage to the photosynthetic apparatus. Under these conditions, PSI cyclic electron transport is increased, contributing to a strong thylakoid lumen acidification, which triggers photoprotective quenching mechanisms to dissipate excess energy (reviewed by, e.g. [50]). Lumen acidification also slows down the oxidation of plastoquinol by the cytochrome (cyt) b_6_f complex, resulting in a reduced PQ-pool and decreased electron transport from PSII; by this so-called “photosynthetic control” mechanism, the accumulation of electrons on PSI is prevented, which can otherwise lead to photodamage [51–53]. Under these conditions, the rate of charge recombination in PSII increases and the rate of O_2_ evolution decreases [54]. The excess reducing power in the photosynthetic electron transport may be alleviated by alternative pathways in *Chlamydomonas*, including the Mehler reaction, the malate shuttle, the plastid terminal oxidase and the flavodiiron-dependent photoreduction reduction pathways (reviewed by [50, 55]). However, these pathways are O_2_-dependent and under hypoxic conditions electron transport to the hydrogenases may represent a more suitable safety valve. To test the possibility that thylakoid lumen acidification in the light may limit linear electron transport and thereby O_2_ and H_2_ production, we treated the cultures with the ionophore carbonylcyanide *p*-trifluoromethoxyphenylhydrazone (FCCP) in the light [56, 57]. Upon the addition of FCCP, H_2_ evolution increased promptly, followed by a transitory increase in O_2_ evolution (Fig. 2i, j), in agreement with the notion that a high ΔpH may limit linear electron flow to the hydrogenases [58].

The fast chl *a* fluorescence (OJIP) transient is a sensitive and widely used indicator of photosynthetic function (e.g. [59]); its F_0_ and F_J_ values can be used to estimate the redox status of the PQ-pool [60, 61]). The latter was evaluated in cell cultures collected directly from serum bottles during H_2_ production experiments, with no dark-adaption prior to the measurement (Additional file 4: Fig. S3). F_0_ and F_J_ values were relatively high in the cultures producing H_2_, indicating that the PQ-pool was in a reduced state. This result corroborates that “photosynthetic control” is an important factor in the establishment of the H_2_ producing conditions.

**Fig. 3:**
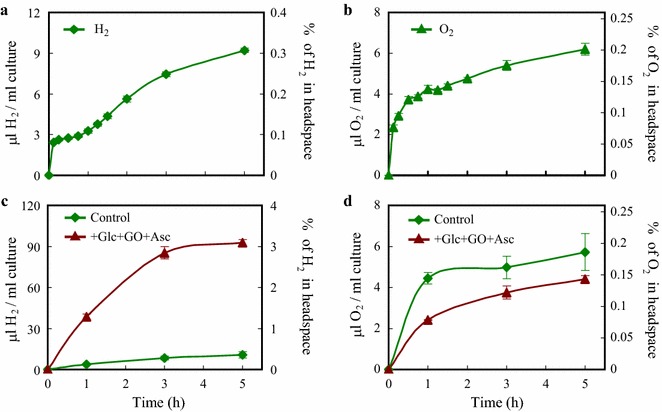
The effect of O_2_ on the H_2_ production of *Chlamydomonas* in acetate-free HS medium induced by dark anaerobic incubation. **a**, **b** Detailed time courses of H_2_ and net O_2_ productions during the initial 5 h of illumination at 320 µmol photons/m^2^/s. **c**, **d** The effects of glucose (Glc, 2 mM), glucose oxidase (GO 0.2 mg/ml) and ascorbate (Asc, 1 mM) on H_2_ and net O_2_ productions, added after the dark anaerobic incubation. In the right *Y* axis, the percentage of the corresponding gases in the headspaces of the cultures are shown. Mean values (± SEM) are each based on 4–8 biological replicates

When measuring the rate of H_2_ production with a higher time resolution in continuous light following dark anaerobic incubation (Fig. 3a), we observed a burst of H_2_ production, as typically occurring in such dark–light transitions [62]. Approximately 2.3 µl H_2_/ml culture was produced in 8 min, corresponding to a rate of 15.76 µmol H_2_/mg chl (a + b)/h. This was followed by a lower H_2_ production rate (1.31 µmol H_2_/mg chl (a + b)/h between 1 and 5 h) during which approx. 0.2% O_2_ accumulated gradually in the gas phase (Fig. 3b). The [Fe–Fe]-type hydrogenases of *C. reinhardtii* have an I_50_ of 0.3–0.4% O_2_ as determined upon a 2-min incubation in vivo [63], with a very minor fraction remaining active at atmospheric O_2_ levels [64]. Thus, a prolonged exposure to 0.2% O_2_ may explain the decreasing rate of H_2_ production.

A way to keep the hydrogenases active may be eliminating the produced O_2_ [65]. As a first approach, we added glucose (Glc), glucose oxidase (GO) and ascorbate (Asc) to the cultures to scavenge O_2_ [60]. The separate additions of Glc, GO and Asc had minor effects on the amount of evolved O_2_ and H_2_ (Additional file 5: Table S2). Upon the combined Glc + GO + Asc treatment, the amount of O_2_ in the headspace was reduced (Fig. 3d), and concomitantly, the amount of H_2_ produced in 3 h increased tenfold, from approx. 8–85 µl H_2_/ml culture (equal to 1.36 and 23.13 µmol H_2_/mg chl (a + b)/h, respectively; Fig. 3c). These values correspond to light-to-H_2_ energy conversion efficiencies of 0.29 and 2.95% in the absence and presence of Glc + GO + Asc, respectively (Table 1, see “Materials and methods” for the calculations). However, this treatment is not a viable option for a long-term H_2_ production because of the Glc requirement and the concomitant reactive oxygen species production [66]; thus we searched for another possibility.

**Table 1:** Light-to-H_2_ energy conversion efficiency in *Chlamydomonas reinhardtii* in HS medium under various conditions

Conditions of H_2_ production	Time of illumination following dark anaerobic incubation (h)				
	0.25	1	3	5	24
Control (HS)					
H_2_ produced (µl/ml)	3.46	3.86	8.41	10.80	27.47
Efficiency (%)	1.43	0.40	0.29	0.22	0.11
+ Glc + GO + Asc					
H_2_ produced (µl/ml)	n.d.	38.57	85.33	92.68	117.04
Efficiency (%)	n.d.	4.00	2.95	1.92	0.51
+ O_2_ absorbent					
H_2_ produced (µl/ml)	3.85	6.73	13.09	19.20	62.10
Efficiency (%)	1.60	0.70	0.45	0.40	0.27

Algal cultures containing 50 µg chl (a + b)/ml and dark-incubated for 4 h were illuminated using cool white fluorescent tubes, at an intensity of 52 W/m^2^, corresponding to approx. 320 µmol photons/m^2^/s. See “Materials and methods” for the calculations on the incident light-to-H_2_ conversion efficiency. The experiments were carried out in HS medium (control) and the effects of a combined glucose (Glc, 2 mM), glucose oxidase (GO, 0.2 mg/ml) and ascorbate (Asc, 1 mM) treatment and the effects of an iron-salt O_2_ absorbent were also assessed

*nd* non-determined

### Preserving hydrogenase activity using an iron-salt based O_2_absorbent

Decreasing the amount of O_2_ in the cultures can be achieved by (i) downregulating PSII activity (e.g. [15, 67]), which is precarious, because PSII is the main source of reducing power; (ii) increasing the respiration:photosynthesis ratio [25], which is limited to low light intensities and requires significant amounts of organic substrates overall leading to low energy conversion efficiencies; and (iii) bacterial respiration [35], with the drawback that it also requires acetate or other organic carbon source. Intense flushing with ultra-pure helium has been adopted to preserve hydrogenase activity [65, 68]; hemoglobin, myoglobin and cobalt chelates have also been used in short-term experiments to remove the evolved O_2_ [69].

**Fig. 4:**
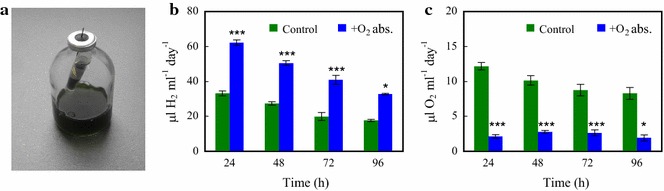
The effect of an iron-salt-based O_2_ absorbent (O20TM) on the H_2_ production of *Chlamydomonas* in acetate-free HS medium induced by dark anaerobic incubation. **a** Photograph of the H_2_-producing *Chlamydomonas* cultures in serum bottles with the iron-salt-based O_2_ absorbent introduced into the headspace. **b**, **c** The effects of the iron-salt-based O_2_ absorbent on the daily H_2_ production and daily O_2_ accumulation in *Chlamydomonas* cultures illuminated continuously at 320 µmol photons/m^2^/s. The cultures were flushed with N_2_ for 10 min every 24 h after determining the gas concentrations in the headspaces of the sealed bottles. Mean values (± SEM) are each based on 11–15 biological replicates. Statistical significance levels are presented relative to the *Chlamydomonas* culture subjected to dark anaerobic incubation in HS medium as *p < 0.05; ***p < 0.001

Here, we opted for using a chemical O_2_ absorbent, a mixture of iron powder and sodium chloride, widely used in the food industry (for details, see “Experimental procedures”). It is highly active (1 g absorbent can absorb up to 13 ml O_2_ at room temperature; e.g. [70]), biologically safe and very cheap (O20TM; http://www.o2zero.com, 20 cc). We placed a small amount (approximately 1.3 g) of O_2_ absorbent into a 2-ml vial and introduced it into the headspace of the serum bottle, above the *Chlamydomonas* culture (Fig. 4a). This system resulted in approx. twofold higher H_2_ productions (Fig. 4b), reaching approx. 200 µl H_2_/ml culture in 96 h [equal to approx. 163.3 µmol H_2_/mg chl (a + b)]. The concentration of O_2_ in the headspace accumulated in 24 h was lowered to approx. 2.1 µl O_2_/ml culture (Fig. 4c), corresponding to 0.07% O_2_ in the headspace.

The light-to-H_2_ energy conversion efficiency was approx. 1.5% both in the absence and presence of the O_2_ absorbent following the anaerobic incubation and 15 min of illumination. However, after 24 h of illumination the light-to-H_2_ energy conversion efficiency decreased to approx. 0.11% in the control samples (HS), whereas it was more than two times higher (approx. 0.27%) when the O_2_ absorbent was present (Table 1). In earlier sulphur deprivation experiments carried out in PBRs and in the presence of acetate, the maximum light-to-H_2_ conversion efficiency was in the range 0.13–3.22%, whereas in nutrient-replete conditions with acetate, it was much lower, approx. 0.1% (see Table 3 in [25], for a comparison on various H_2_ producing conditions).

We have also compared the H_2_ production yields at three different chl concentrations. In the absence of the O_2_ absorbent, the total amounts of H_2_ produced in 96 h were approx. 50, 90 and 105 µl/ml culture at 15, 30 and 50 µg chl (a + b)/ml culture, respectively. In the presence of the O_2_ absorbent, productivities increased to approx. 120, 210 and 227 µl H_2_/ml culture, respectively (Additional file 6: Fig. S4). Thus, reducing the chl content from 50 to 15 µg chl (a + b)/ml improved H_2_ production on a chl basis even in the absence of the O_2_ absorbent (from 2.1 to 3.3 ml H_2_/mg chl (a + b), respectively).

Daily N_2_ flushing was applied to periodically remove the produced gases. To test whether this was essential, we carried out an experiment without gas removal. In the absence of the O_2_ absorbent and without N_2_ flushing, the amount of H_2_ produced was reduced by about 50%. However, when O_2_ absorbent was present, the amounts of H_2_ were in the same range with our without N_2_ flushing (Additional file 7: Fig. S5).

**Fig. 5:**
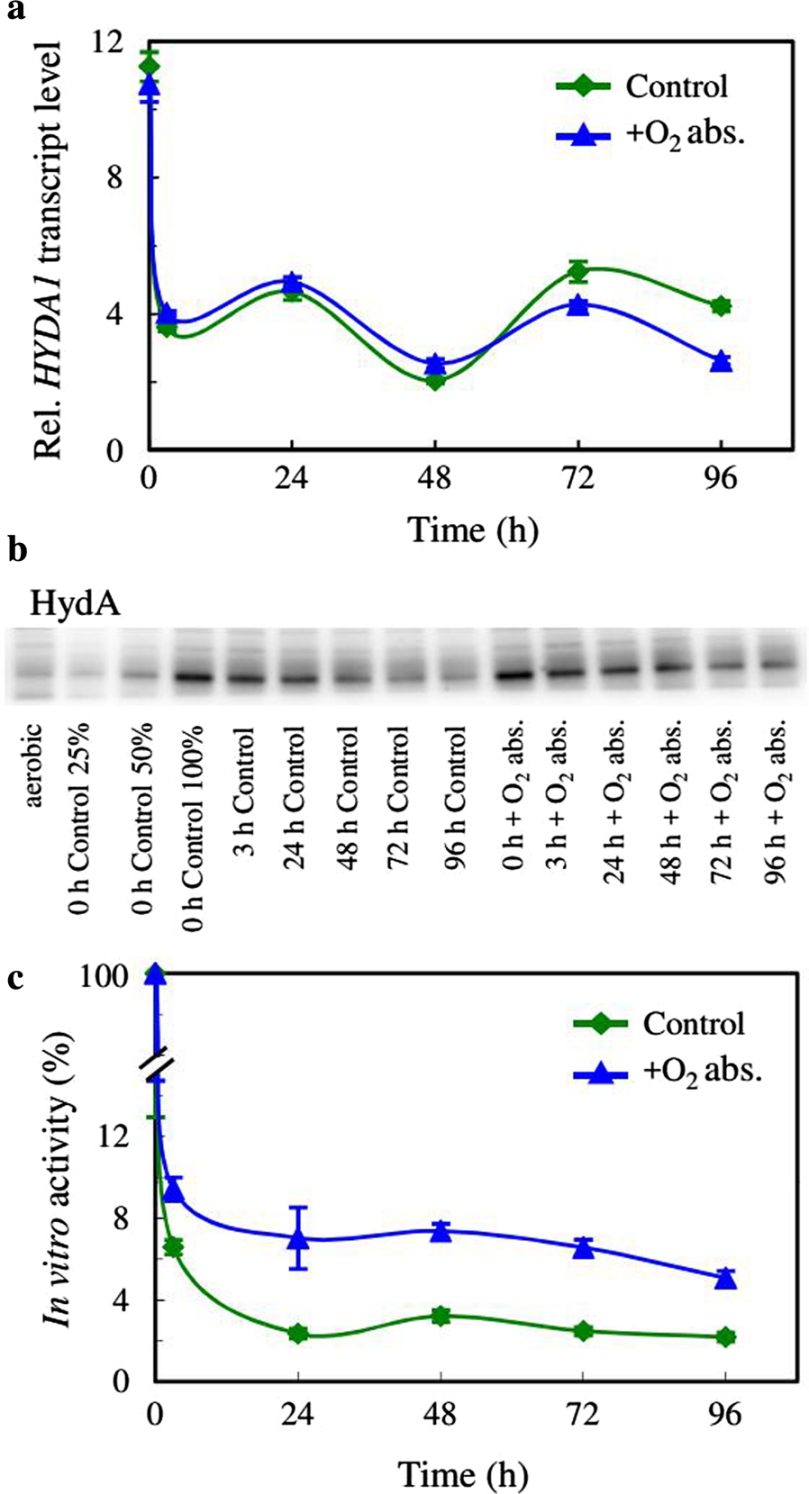
The effects of an iron-salt-based O_2_ absorbent on the hydrogenase activity of *Chlamydomonas* in acetate-free HS medium induced by dark anaerobic incubation. **a** *HYDA1* expression level, expressed relative to the aerobically cultured controls in the presence and absence of the O_2_ absorbent. **b** The amount of HydA, determined by western blot analysis in the presence and absence of the O_2_ absorbent; samples of 2 µg chl (a + b) were loaded and the second to fourth lanes (25, 50, 100% of 0 h control) are for approximate quantitation of HydA. The 0 h control samples were collected directly after the 4-h dark anaerobic incubation. **c** The in vitro hydrogenase activity in the presence and absence of the O_2_ absorbent, expressed as a percentage of the activity of cells subjected to dark anaerobic incubation for 3 h, which had an in vitro hydrogenase activity of approx. 500 µmol H_2_/mg chl (a + b)/h. Mean values (± SEM) are each based on 3 or 4 biological replicates

Next, we assessed the expression and the activity of hydrogenases in the presence and absence of the O_2_ absorbent. During the 4-h dark anaerobic incubation period, the *HYDA1* transcript level increased approx. 11-fold, as determined by quantitative RT-PCR analysis (Fig. 5a), which was followed by a decrease both in the presence and absence of the O_2_ absorbent, possibly due to the strong inhibitory effect of O_2_ on *HYDA1* expression [12, 13]. The amount of HydA enzyme as determined semi-quantitatively by western blot analysis, also showed a strong upregulation upon the 4-h dark anaerobic incubation period. Later on, the amount of HydA decreased, which was attenuated by the O_2_ absorbent (Fig. 5b). Following the dark anaerobic incubation, the in vitro hydrogenase activity, as determined in the presence of reduced methylviologen as an electron donor, was approx. 500 µmol H_2_/mg chl (a + b)/h, in agreement with literature data ([24]; see “Experimental procedures” for details). Upon 4 h of illumination, the in vitro hydrogenase activity decreased to approx. 6 and 9% of the original activity in the absence and presence of the O_2_ absorbent, respectively (Fig. 5c), highlighting the extreme O_2_ sensitivity of hydrogenases [63]. Following this rapid decline, there was a slower phase of inactivation, and by the end of the experiment 2% of the original in vitro activity remained in the absence of the O_2_ absorbent, while 5% remained in its presence (Fig. 5c).

### Photosynthetic activity during H_2_ production

**Fig. 6:**
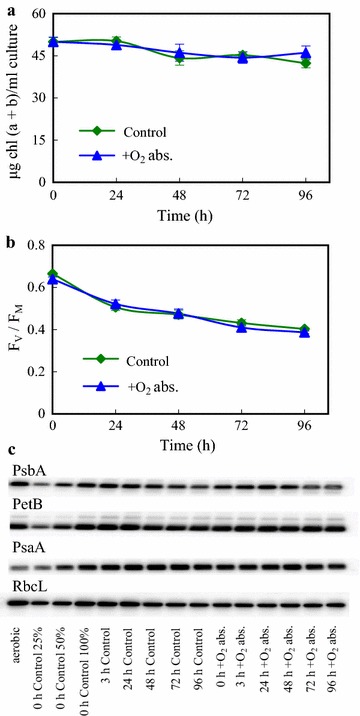
Characterisation of the photosynthetic apparatus of *Chlamydomonas* during H_2_ production in acetate-free HS medium induced by dark anaerobic incubation in the absence and presence of an iron-salt-based O_2_ absorbent. **a** Changes in chl (a + b) content during the H_2_-producing period. **b** The F_V_/F_M_ fluorescence parameter determined based on OJIP transients. **c** Western blot analysis for the semi-quantitative determination of PsbA, PetB, PsaA and RbcL contents; samples of 2 µg chl (a + b) were loaded and the second to fourth lanes (25, 50, 100% of 0 h control) are for approximate quantitation of the proteins. The 0 h control samples were collected directly after the 4-h dark anaerobic incubation. Mean values (± SEM) are each based on 3–6 biological replicates

The maintainability of our system was tested by characterization of the photosynthetic apparatus during the 96-h H_2_-producing period. The chl (a + b) content decreased only by approx. 10% during the 96-h experiment (Fig. 6a). The *F*_V_/*F*_M_ value, an indicator of PSII efficiency, slowly decreased during the 96-h H_2_ producing period, but remained relatively high (above 0.4) both in the presence and absence of the O_2_ absorbent (Fig. 6b).

The amount of PsbA (reaction center protein of PSII), PetB (a subunit of the cytb_6_/f complex) and PsaA (reaction center protein of PSI) remained largely unaltered during the 96-h H_2_-producing period, both in presence and absence of the O_2_ absorbent, as determined by western blot analysis (Fig. 6c); the amount of the large Rubisco subunit, RbcL, showed a moderate decrease (Fig. 6c). These findings are in strong contrast to the sulphur deprivation protocol in TAP medium, where the amount of Rubisco and PsbA strongly diminish within 48 h. By the end of a 4–6-day period of sulphur deprivation most photosynthetic complexes are degraded and the cells eventually die [15, 17, 41, 71].

**Fig. 7:**
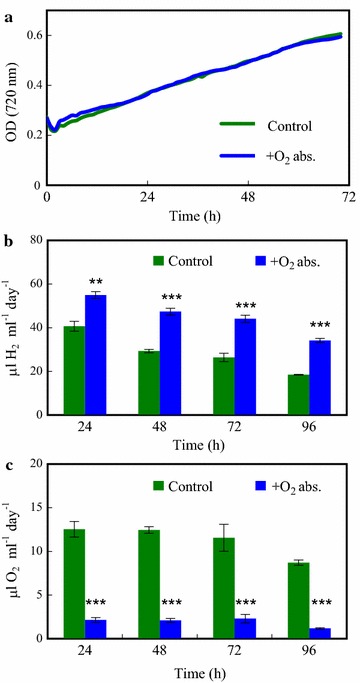
Growth of *Chlamydomonas* cultures following the H_2_ production phase and a second round of H_2_ production induced by dark anaerobic incubation in HS medium. **a** Culture growth in HS medium bubbled with sterile air containing 1% CO_2_, as determined by measuring optical density at 720 nm, following the first round of H_2_ production. During the growth phase, no O_2_ absorbent was present, only during the preceding H_2_ production phase. **b**, **c** H_2_ production and O_2_ accumulation in cultures subjected to a second round of dark anaerobic incubation in the presence and absence of an iron-salt-based O_2_ absorbent. The cultures were flushed every 24 h with N_2_ after determining the gas concentrations in the headspaces of the sealed bottles. Mean values (± SEM) are each based on 4 biological replicates. Statistical significance levels are presented relative to the *Chlamydomonas* culture subjected to dark anaerobic incubation in HS medium without O_2_ absorbent as ***p < 0.001

Since our cells remained photosynthetically active, we attempted recovering and reusing the cultures after the H_2_-producing period. Cultures were diluted with HS medium to 6 µg chl (a + b), transferred into a multi-well cultivation instrument and sparged with air containing 1% CO_2_ for 72 h. The algal cultures showed discernible growth in the HS medium and the cell density doubled within approx. 24 h, as estimated based on optical density (Fig. 7a). When these cells were subjected to a second dark anaerobic incubation period in HS media (as described above), H_2_ production occurred with yields similar to the first cycle (cf. Figs. 4b, c, 7b, c).

## Discussion

The potential energy conversion efficiency from sunlight to H_2_ by green algae is in the range of 10–13% [14, 72]. However, in nature H_2_ production lasts only for a few minutes due to the inhibition of hydrogenases by the evolved O_2_ [20, 27]. Early studies on algal H_2_ production based on dark anaerobic incubation were typically unable to sustain the initial high rates of H_2_ production for more than a few hrs, if not resorting to continuous flushing with helium [31, 65, 68]. Later, sulphur deprivation became the method of choice to induce long-term H_2_ production [15, 17, 42]. However, sulphur deprivation has several drawbacks which impede its industrial application [20, 72]: the procedure requires several washing steps; H_2_ production starts with a delay of about 2 days; it is largely dependent on acetate (H_2_ production can be induced under photoautotropic conditions as well, but with a much lower efficiency [18–20]); it necessitates the inactivation of PSII; and it results in the degradation of the photosynthetic machinery. Recovery following the terminal phase of H_2_ production by re-additions of sulphur was incomplete and could be performed only a few times [73].

The future of this biotechnology relies on the development of a novel approach at least as efficient as the sulphur deprivation procedure, and which could solve most of the present issues limiting its applicability. Here we report on the establishment of a photoautotrophic and sustainable H_2_ production system in *C. reinhardtii*, and demonstrate the applicability of algal cells as whole-cell catalysts for H_2_ production. This new protocol shares the early approach by Gaffron and Rubin [31] to induce hydrogenase activity by dark anaerobic treatment and keep the CBB cycle inactive by substrate limitation. As an important addition, the protocol applies a simple O_2_ absorbent that preserve hydrogenase activity for several days (Fig. 4). This protocol has fundamental advantages relative to the earlier methods, namely that (i) following a few hours of anaerobic dark incubation, H_2_ production starts promptly upon illumination (Fig. 1b); (ii) as opposed to sulphur deprivation, it does not require media exchange (Fig. 7); (iii) it does not depend on starch degradation and does not require acetate, thus it is photoautotrophic; (iv) because no organic carbon source is required, the risk of bacterial contamination is low; (v) the cultures remain photosynthetically active during the H_2_ production phase (Fig. 6) and they can be easily recovered afterwards (Fig. 7); (vi) it is based on linear electron transport and the electrons originate mostly from the water-splitting activity of PSII, as demonstrated by a DCMU-treatment (Fig. 2), and has relatively high light-to-H_2_ energy conversion efficiencies (Table 1); (vii) during the growth phase, CO_2_, an industrial by-product, can be utilized; and, (viii) it can make use of relatively high light intensities (here, approx. 320 µmol photons/m^2^/s).

The maximum H_2_ production yield achieved using this protocol was approx. 200 µl H_2_/ml culture in 96 h, which is almost four times higher than the yield of sulphur-deprived cultures at equal chl content and illumination conditions (compare Figs. 1d, 4b), and it is in the same range as observed earlier for sulphur deprivation experiments using PBRs with even illumination [42], but having the drawbacks listed above. We expect the yield of H_2_ production achievable upon anaerobic induction in minimal media to be further improved using advanced PBR designs, including optimized gas-to-liquid ratio, illumination and mixing conditions and efficient removal of the produced gases. There is also a high potential in applying this protocol to various photosynthetic mutants possessing, e.g. truncated light-harvesting antennae [74], or a high PsbA protein content [75]. Cyclic electron transport competes for the electrons with HydA (reviewed by [9]), thus its downregulation may entail a further increase in H_2_ production under our conditions as well.

Keeping the CBB cycle inactive was achieved by substrate limitation; it has been shown earlier that the CBB cycle represents a competing pathway for H_2_ production (e.g. [31, 47, 49, 68]) and that redirecting the electrons towards HydA from FNR may enhance the rate of H_2_ production [76, 77].

The present findings show that by imposing substrate limitation on the CBB cycle, the electrons are largely transferred to HydA, with the lack of carbon sources facilitating the establishment of hypoxia. The effects of CO_2_ and FCCP additions (Fig. 2) and the relatively reduced PQ-pool (Additional file 4: Fig. S3) indicate that the mechanism occurs by “photosynthetic control” [51, 54, 78]: since the hydrogenases are less effective at accepting electrons than the CBB cycle, the lumen is acidified and the photosynthetic electron transport is decelerated at the cytb_6_f complex. This results in a reduced PQ-pool, which entails a high charge recombination rate in PSII, resulting in diminished O_2_ evolution (Fig. 2i, j).

Another key factor to reach a sustained H_2_ production is to protect the hydrogenases from O_2_, which may also shift the balance between O_2_ and H_2_ production, established by the above-mentioned “photosynthetic control”. We applied an iron-salt-based O_2_ absorbent, which decreased the O_2_ concentration in the headspace below 0.1%. This very low concentration of O_2_ was still inhibitory for hydrogenases (Fig. 5), thus it is desirable to test even more advanced materials in the future, as for instance crystalline salts of cationic multimetallic cobalt complexes [79]. By further decreasing the O_2_ level, we expect that hydrogenase activity would be better preserved and act as a more effective electron sink; as a result, lumen acidification and “photosynthetic control” will be attenuated, and the yield of H_2_ production further increased. Engineering hydrogenases to tolerate a few percent of O_2_ [14] could also be a successful strategy to further increase the efficiency of H_2_ production.

## Experimental procedures

### Algal growth conditions and H_2_ production

*Chlamydomonas reinhardtii* CC124 strain was grown initially at 22 °C in 250 ml Erlenmeyer flasks containing 50 ml Tris–acetate–phosphate (TAP) medium shaken at 120 rpm in an algal growth chamber under continuous illumination of 80–90 µmol photons/m^2^/s PAR (measured by a LI-250A light meter equipped with a quantum sensor), provided by white fluorescent tubes.

After 72 h of cultivation, the cells were transferred to high-salt (HS) medium, HS supplemented with acetate (HSA), Tris–phosphate (TP) or Tris–acetate–phosphate (TAP) media (http://www.chlamycollection.org/methods/media-recipes/) and the chl content was set at 50 µg chl (a + b)/ml [80], corresponding to approx. 15 million cells/ml as determined by a Millipore Scepter cell counter (described in [81]). In one experiment, 15 and 30 µg chl (a + b)/ml was also set. For H_2_ production, 30 ml culture was placed in a 120-ml serum bottle and sealed off with rubber septa under sterile conditions. For dark anaerobic incubation, the gas phase of the bottle was flushed with N_2_ gas for 10 min and kept in the dark for 4 h, during which the vials were flushed twice more with N_2_. To produce sulphur-deprived cultures, the cells were washed five times with sulphur-free TAP medium (centrifugation at 1000*g*, at 24 °C for 5 min, see also [41]) and the chl content was set at 50 µg chl (a + b)/ml. Following these steps, the cultures were placed under T8 cool white fluorescent light tubes (Sylvania luxline plus), providing approximately 52 W/m^2^ at the level of the cultures as determined by a Spectra-Physics 404 Power meter; this light intensity corresponded to approx. 320 µmol photons/m^2^/s PAR as measured by a LI-250A light meter equipped with a quantum sensor. The cultures were illuminated continuously and kept at 26 °C for 96 h. For a general scheme of the experiments, see Additional file 1: Fig. S1.

In the regeneration experiment following H_2_ production, a Multi-Cultivator MC 1000-OD instrument (Photon Systems Instruments, Brno, Czech Republic) was used. At the start of cultivation, the chl content was set at 6 µg chl (a + b)/ml in HS medium. The cells were grown in HS medium at 23 °C, 80 µmol photons/m^2^/s in continuous light provided by white LEDs for 72 h and the cultures were bubbled with sterile air containing 1% CO_2_. Following this, the cultures were subjected to a second round of dark anaerobic incubation in HS medium, as described above.

### Chemical treatments

In separate experiments, 20 µM 3-(3′,4′-dichlorophenyl)-1,1-dimethylurea (DCMU) dissolved in dimethyl sulfoxide (DMSO, 100 mM stock solution), 1 mM potassium cyanide (KCN) and 1 mM salicylhydroxamic acid (SHAM), 2% v/v CO_2_, 10 mM glycolaldehyde (GA) dissolved in water (1.5 M stock solution) and 2 µM carbonylcyanide* p*-triflouromethoxyphenylhydrazone (FCCP) dissolved in HS medium (20 mM stock solution) were added to the cultures 3 h after the onset of light and start of H_2_ production. To eliminate the produced O_2_, 2 mM glucose (Glc, 1 M stock solution), 0.2 mg/ml glucose oxidase (GO, 30 mg/ml stock solution) [82] and 1 mM ascorbate (Asc, 1 M stock solution) were added to the cultures before the start of dark anaerobic incubation.

An iron-salt-based, non-cytotoxic O_2_ absorbent (O20_TM_; http://www.o2zero.com, 20 cc) was used to eliminate O_2_ from the headspace of the serum bottles during the 96-h H_2_ production phase. To this end, 1.3 g of O_2_ absorbing material was placed into a 2 ml-vial, which was left open and introduced into the headspace of the serum bottles; the algal culture did not get into direct contact with the O_2_ absorbent (Fig. 4a).

### Determination of net H_2_ and O_2_ production by gas chromatography

The net amounts of H_2_ and O_2_ produced by the cells were determined by taking 250 µl aliquot from the gas phase of the cultures with a gas tight microsyringe. These samples were injected manually into an Agilent 6890 N gas chromatograph (GC) equipped with a HP-PLOT Molesieve 5 Å column (30 m × 0.53 mm × 0.25 µm) and a TCD detector. The oven temperature was 30 °C. The carrier gas was argon, and a linear velocity of 115 cm/s was used. The bottles were flushed with N_2_ gas every 24 h following the determination of gas production.

### In vitro hydrogenase activity assay

In vitro hydrogenase activity was measured after the dark anaerobic incubation and during the course of H_2_ production in the light, as described in [24]. The assay was carried out in 13.5-ml serum vials at 37 °C and the reaction mixture consisted of 1 ml of 100 mM potassium phosphate buffer, pH 6.8, 80 µl of deionized water, 200 µl of 10% Triton X-100, 20 µl of 1 M methylviologen, 200 µl of anaerobic 1 M sodium dithionite and 200 µl of algal culture. The H_2_ concentration in the headspace was measured by GC every 15 min and fitted with linear regression. Results are the mean value of tests performed in at least four replicates. Following a 4-h dark anaerobic incubation the hydrogenase activity was approx. 500 µmol H_2_/mg chl (a + b)/h, in agreement with [24]. The data are presented as percentages of this original (maximum) activity.

### RNA isolation and qRT-PCR analysis to assess HYDA1 transcript level

For RNA isolation, 1 ml culture, containing approximately 50 µg chl (a + b), was collected and the Direct-Zol RNA kit was used, following the recommendations of the manufacturer (ZymoResearch). To remove contaminating DNA from the samples, the isolated RNA was treated with DNaseI (ZymoResearch). RNA integrity was checked on a 1% (w/v) MOPS gel. Reverse transcription was primed with oligo dT using 1 µg of total RNA and SuperScript III reverse transcriptase (Life Technologies). To confirm the absence of DNA contaminations, an aliquot of the RNA sample was used without reverse transcriptase.

Real-time qPCR analysis was performed using an Applied Biosystems Prism 7900HT Fast Real Time PCR System using HOT FIREPol^®^ EvaGreen^®^ qPCR Mix Plus (ROX) (Solis BioDyne). Primers were designed using the NCBI Primer Blast Tool (http://www.ncbi.nlm.nih.gov/tools/primer-blast/). The melting temperature was 60 °C and the amplicon length was between 100 and 130 bp. To ensure correct normalization of the *HYDA1* (Cre03.g199800) transcript level, three reference genes showing stable expression during H_2_ production were used, namely *bTUB2* (Cre12.g549550), *ACTIN* (Cre13.g603700) and *UBQ* (XP_001694320). The primers for *HYDA1* were GGCGAGTGGGACAATCCAAT and TGCCCGTGAACAGCTCATAG; for the reference genes, see [83]. The data are presented as fold-change in mRNA transcript abundance of *HYDA1*, normalized to the average of the three reference genes, and relative to the control sample (cultures grown in TAP medium under normal growth conditions). The analysis was carried out with three technical replicates and two or three biological replicates; the standard error was calculated based on the range of fold-change by calculating the minimum and the maximum of the fold-change using the standard deviations of the ΔΔCt.

### Fast chl a fluorescence (OJIP) measurements

Fluorescence measurements were carried out with a Handy-PEA instrument (Hansatech Instruments Ltd, UK). For F_V_/F_M_ measurements, *C. reinhardtii* cultures were dark-adapted for 15 min and then 3 ml of cell suspension (50 µg chl (a + b)/ml) was filtered onto a Whatman glass microfibre filter (GF/B) that was placed in a Handy-PEA leaf clip. For the assessment of the PQ-pool redox status, the cultures were measured immediately after taking them from the serum bottles, without any dark adaptation. The algal sample was illuminated with continuous red light (3500 µmol photons/m^2^/s, 650 nm peak wavelength; the spectral half-width was 22 nm; the light emitted by the LEDs is cut off at 700 nm by a NIR short-pass filter). The light was provided by an array of three light-emitting diodes focused on a circle of 5 mm diameter of the sample surface. The first reliably measured point of the fluorescence transient is at 20 µs, which was taken as *F*_0_.

### Western blot analysis and determination of starch content

Two ml of culture were collected at each time-point and the analyses were carried out as described in [41] with slight modifications.

### Calculation of light-to-H_2_ energy conversion efficiency

Efficiency of light-to-H_2_ energy conversion (*η*_c_) was calculated as the ratio of the rate of chemical energy production (k_H_ HHV_H_) to the incident light power (*E*_s_*A*) (similarly to [84]). The rate of chemical energy production (as H_2_) is expressed as the product of the rate of H_2_ evolution (k_H_) measured in mol/s units and the higher heating value of H_2_ (HHV_H_ = 286,000 J/mol).

The incident light power (*E*_s_*A*) was calculated from the incident light intensity (E_s_, in W/m^2^) and the illuminated area (*A*).

$$\eta_{\text{c}} \, = \,{\text{k}}_{\text{H}}\,{\text{HHV}}_{\text{H}} /E_{\text{s}} A$$

Light was provided by T8 cool white fluorescent tubes. Its incident intensity was 52 W/m^2^ as determined using a Spectra-Physics 404 Power meter. The temperature during H_2_ production was 298 K, the diameter of the serum bottles was 0.048 m and the irradiated area was 0.0018 m^2^.

### Statistics

The presented data are based on at least three independent experiments. When applicable, averages and standard errors (SEM) were calculated. Statistical significance was analysed using Student’s *t* test and the significance level are presented as: *p < 0.05; **p < 0.01; ***p < 0.001.

## Additional files

**Additional file 1: Figure S1.** General scheme of the H_2_ production experiment induced by dark anaerobic incubation. Notes: (1) HS media was used in most experiments, except for Fig. 1, where TAP, TP, HSA, HS and TAP-S media were compared. (2) Before the start of dark anaerobic incubation, O_2_ absorbent was placed in the headspaces of the cultures (Figs. 4, 5, 6, Additional file 6: Fig. S4, Additional file 7: Fig. S5). (3) Chemicals were added after 3 h of illumination (Fig. 2) or at the beginning of illumination (Fig. 3). (4) Sampling of the cultures at various time intervals (Figs. 5, 6, Additional file 2: Fig. S2, Additional file 4: Fig. S3, Additional file 6: Fig. S4, Additional file 7: Fig. S5). (5) The cultures were regenerated following the 96-h H_2_ production using HS medium and CO_2_ bubbling; afterwards, a second round of H_2_ production was carried out (Fig. 7).

**Additional file 2: Figure S2.** Starch content of *Chlamydomonas* cultures subjected to dark anaerobic incubation followed by continuous illumination at 320 µmol photons/m^2^/s in acetate-free HS medium. Time 0 is the time point when the cultures were transferred to the light.

**Additional file 3: Table S1.** The percentage of CO_2_ in the headspaces of sealed cultures of Chlamydomonas cultures subjected to dark anaerobic incubation of 4 h in HS medium followed by continuous illumination of 320 µmol photons/m^2^/s, as determined using gas chromatography. Mean values (± SEM in parentheses) are each based on 4–6 biological replicates. bld: below detection limit of 0.01%.

**Additional file 4: Figure S3.** Fast chl *a* fluorescence (OJIP) transients of *Chlamydomonas* cultures subjected to dark anaerobic incubation followed by continuous illumination at 320 µmol photons/m^2^/s in acetate-free HS medium. Time 0 is the time point when the cultures were transferred to the light. For the fluorescence measurements, the cultures were measured immediately after taking them from the serum bottles, without any dark adaptation.

**Additional file 5: Table S2.** The effects of the separate and combined additions of glucose (Glc, 2 mM), glucose oxidase (GO, 0.2 mg/ml) and ascorbate (Asc, 1 mM) on the net H_2_ and O_2_ productions of Chlamydomonas cultures subjected to dark anaerobic incubation of 4 h in HS medium followed by continuous illumination of 320 µmol photons/m^2^/s, as determined in the headspaces of sealed cultures using gas chromatography. Mean values (± SEM in parentheses) are each based on 4 to 8 biological replicates.

**Additional file 6: Figure S4.** H_2_ production yields (a, c) and O_2_ concentrations in the headspaces of the serum bottles (b, d) at 15, 30 and 50 µg chl (a + b)/ml culture in the absence (a, b) and the presence (c, d) of an iron-salt-based O_2_ absorbent. Apart from changing the chl concentrations, the experimental conditions are identical to Fig. 4. The cultures were flushed with N_2_ for 10 min every 24 h after determining the gas concentrations in the headspaces of the sealed bottles. Mean values (± SEM) are each based on 5 to 6 biological replicates.

**Additional file 7: Figure S5.** Continuous H_2_ production (a) and O_2_ accumulation (b) at 50 µg chl (a + b)/ml culture in the absence and the presence of an iron-salt-based O_2_ absorbent. Apart from omitting the daily N_2_ flushing, the experimental conditions are identical to Fig. 4. Mean values (± SEM) are each based on 5–6 biological replicates.
