# Supplementary figures and images for: Water-splitting-based, sustainable and efficient H2 production in green algae as achieved by substrate limitation of the Calvin–Benson–Bassham cycle
Source: Biotechnol Biofuels. 2018 Mar 19;11:69. doi: 10.1186/s13068-018-1069-0 (PMC5858145; doi:10.1186/s13068-018-1069-0)

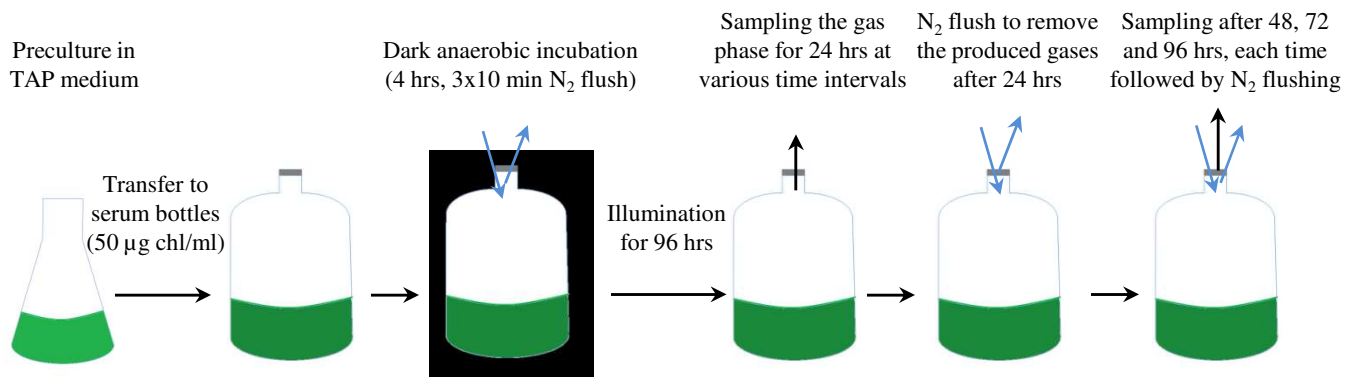

Supplement: Supplementary file 1 — Additional file 1: Figure S1. General scheme of the H2 production experiment induced by dark anaerobic incubation. Notes: (1) HS media was used in most experiments, except for Fig. 1, where TAP, TP, HSA, HS and TAP-S media were compared. (2) Before the start of dark anaerobic incubation, O2 absorbent was placed in the headspaces of the cultures (Figs. 4, 5, 6, Additional file 6: Fig. S4, Additional file 7: Fig. S5). (3) Chemicals were added after 3 h of illumination (Fig. 2) or at the beginning of illumination (Fig. 3). (4) Sampling of the cultures at various time intervals (Figs. 5, 6, Additional file 2: Fig. S2, Additional file 4: Fig. S3, Additional file 6: Fig. S4, Additional file 7: Fig. S5). (5) The cultures were regenerated following the 96-h H2 production using HS medium and CO2 bubbling; afterwards, a second round of H2 production was carried out (Fig. 7). [file 13068_2018_1069_MOESM1_ESM.pdf]

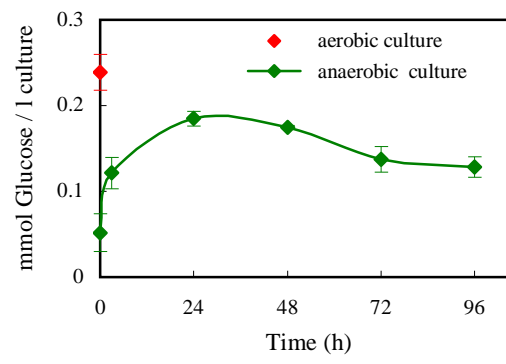

Supplement: Supplementary file 2 — Additional file 2: Figure S2. Starch content of Chlamydomonas cultures subjected to dark anaerobic incubation followed by continuous illumination at 320 µmol photons/m2/s in acetate-free HS medium. Time 0 is the time point when the cultures were transferred to the light. [file 13068_2018_1069_MOESM2_ESM.pdf]

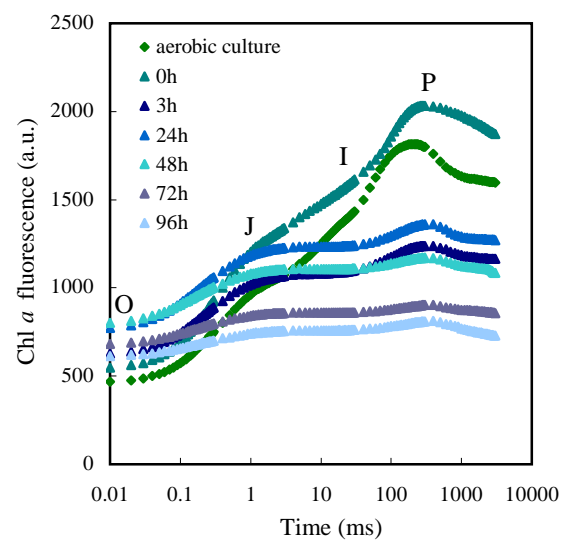

Supplement: Supplementary file 4 — Additional file 4: Figure S3. Fast chl a fluorescence (OJIP) transients of Chlamydomonas cultures subjected to dark anaerobic incubation followed by continuous illumination at 320 µmol photons/m2/s in acetate-free HS medium. Time 0 is the time point when the cultures were transferred to the light. For the fluorescence measurements, the cultures were measured immediately after taking them from the serum bottles, without any dark adaptation. [file 13068_2018_1069_MOESM4_ESM.pdf]

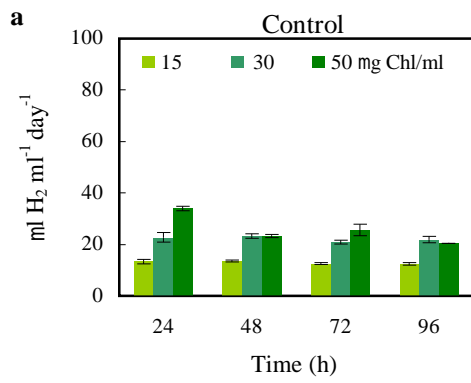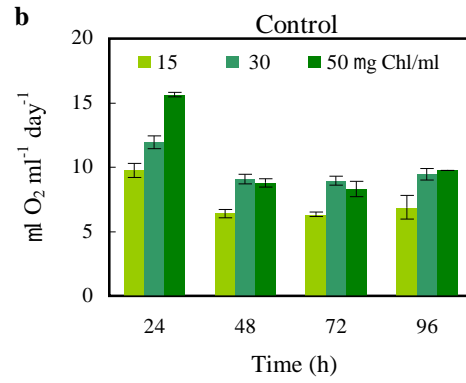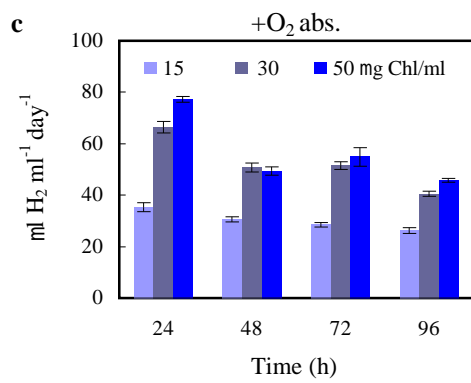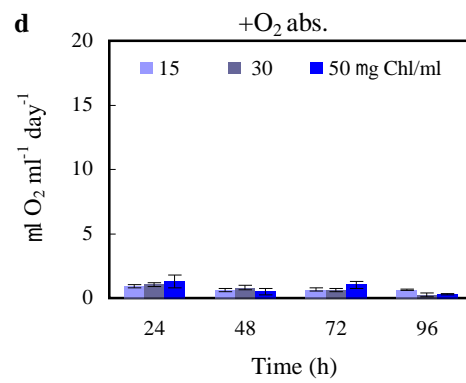

Supplement: Supplementary file 6 — Additional file 6: Figure S4. H2 production yields (a, c) and O2 concentrations in the headspaces of the serum bottles (b, d) at 15, 30 and 50 µg chl (a + b)/ml culture in the absence (a, b) and the presence (c, d) of an iron-salt-based O2 absorbent. Apart from changing the chl concentrations, the experimental conditions are identical to Fig. 4. The cultures were flushed with N2 for 10 min every 24 h after determining the gas concentrations in the headspaces of the sealed bottles. Mean values (± SEM) are each based on 5 to 6 biological replicates. [file 13068_2018_1069_MOESM6_ESM.pdf]

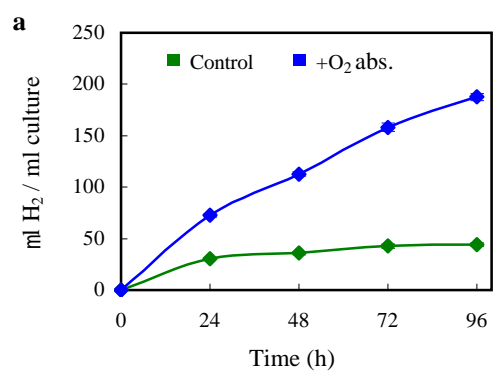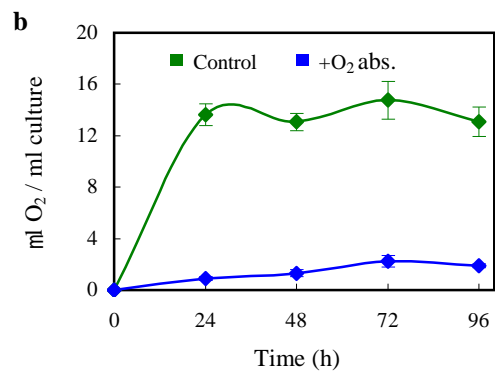

Supplement: Supplementary file 7 — Additional file 7: Figure S5. Continuous H2 production (a) and O2 accumulation (b) at 50 µg chl (a + b)/ml culture in the absence and the presence of an iron-salt-based O2 absorbent. Apart from omitting the daily N2 flushing, the experimental conditions are identical to Fig. 4. Mean values (± SEM) are each based on 5–6 biological replicates. [file 13068_2018_1069_MOESM7_ESM.pdf]
