# Supplementary material for: Water-splitting-based, sustainable and efficient H2 production in green algae as achieved by substrate limitation of the Calvin–Benson–Bassham cycle
Source: Biotechnol Biofuels. 2018 Mar 19;11:69. doi: 10.1186/s13068-018-1069-0 (PMC5858145; doi:10.1186/s13068-018-1069-0)
Supplement: Supplementary file 3 — Additional file 3: Table S1. The percentage of CO2 in the headspaces of sealed cultures of Chlamydomonas cultures subjected to dark anaerobic incubation of 4 h in HS medium followed by continuous illumination of 320 µmol photons/m2/s, as determined using gas chromatography. Mean values (± SEM in parentheses) are each based on 4–6 biological replicates. bld: below detection limit of 0.01%. [file 13068_2018_1069_MOESM3_ESM.docx]

**Table S1.** The percentage of CO_2_ in the headspaces of sealed cultures of Chlamydomonas cultures subjected to dark anaerobic incubation of 4 h in HS medium followed by continuous illumination of 320 µmole photons m^-2^s^-1^, as determined using gas chromatography. Mean values (±SEM in parentheses) are each based on 4 to 6 biological replicates. bld: below detection limit of 0.01%.

| Time of illumination (h) | % of CO_2_ in headspace | |
| --- | --- | --- |
|  | -CO_2_ | +CO_2_ |
| 0 | bld. | 2 |
| 0.25 | bld. | 0.623 (±0.05) |
| 1 | bld. | 0.020 (±0.01) |
| 2 | bld. | 0.104 (±0.02) |
| 3 | bld. | 0.144 (±0.04) |
| 24 | bld. | bld. |
