# Supplementary material for: Water-splitting-based, sustainable and efficient H2 production in green algae as achieved by substrate limitation of the Calvin–Benson–Bassham cycle
Source: Biotechnol Biofuels. 2018 Mar 19;11:69. doi: 10.1186/s13068-018-1069-0 (PMC5858145; doi:10.1186/s13068-018-1069-0)
Supplement: Supplementary file 5 — Additional file 5: Table S2. The effects of the separate and combined additions of glucose (Glc, 2 mM), glucose oxidase (GO, 0.2 mg/ml) and ascorbate (Asc, 1 mM) on the net H2 and O2 productions of Chlamydomonas cultures subjected to dark anaerobic incubation of 4 h in HS medium followed by continuous illumination of 320 µmol photons/m2/s, as determined in the headspaces of sealed cultures using gas chromatography. Mean values (± SEM in parentheses) are each based on 4 to 8 biological replicates. [file 13068_2018_1069_MOESM5_ESM.docx]

**Table S2.** The effects of the separate and combined additions of glucose (Glc, 2 mM), glucose oxidase (GO, 0.2 mg/ml) and ascorbate (Asc, 1 mM) on the net H_2_ and O_2_ productions of Chlamydomonas cultures subjected to dark anaerobic incubation of 4 h in HS medium followed by continuous illumination of 320 µmole photons m^-2^s^-1^, as determined in the headspaces of sealed cultures using gas chromatography. Mean values (±SEM in parentheses) are each based on 4 to 8 biological replicates.

|  |  | Amount of H_2_ and O_2_ produced (µl/ml culture) | | |
| --- | --- | --- | --- | --- |
| Conditions of  H_2_ production |  | Time of illumination following  a 4-h anaerobic incubation (h) | | |
|  |  | 1 | 3 | 5 |
| Control (HS) | H_2_ | 4.57 (±0.24) | 9.19 (±0.85) | 13.71 (±1.09) |
|  | O_2_ | 4.04 (±0.83) | 5.08 (±1.05) | 6.28 (±1.46) |
| +Glc+GO+Asc | H_2_ | 38.57 (±2.04) | 85.32 (±4.49) | 92.68 (±2.41) |
|  | O_2_ | 2.41 (±0.02) | 3.75 (±0.32) | 4.40 (±0.17) |
| +Glc | H_2_ | 6.05 (±0.48) | 10.75 (±0.73) | 14.57 (±1.11) |
|  | O_2_ | 4.64(±0.08) | 6.07 (±0.17) | 7.63 (±0.76) |
| +GO | H_2_ | 8.18(±0.46) | 12.71 (±2.26) | 20.11 (±0.46) |
|  | O_2_ | 6.34 (±0.17) | 8.39 (±0.35) | 10.23 (±0.81) |
| +Asc | H_2_ | 7.78 (±0.42) | 13.49 (±0.72) | 18.58 (±0.85) |
|  | O_2_ | 4.62 (±0.40) | 6.25 (±0.40) | 7.65 (±0.37) |
